# Platelet-rich plasma for long bone healing

**DOI:** 10.1590/S1679-45082013000100023

**Published:** 2013

**Authors:** Mário Lenza, Silvia de Barros Ferraz, Dan Carai Maia Viola, Oscar Fernando Pavão dos Santos, Miguel Cendoroglo, Mario Ferretti

**Affiliations:** 1 Hospital Israelita Albert Einstein, São Paulo, SP, Brazil

**Keywords:** Platelet-rich plasma, Fracture healing, Bone fractures, Pseudoarthrosis, Fracture fixation

## Abstract

**Objective:**

To evaluate effectiveness of the use of platelet-rich plasma as coadjuvant for union of long bones.

**Methods:**

The search strategy included the Cochrane Library (via Central) and MEDLINE (via PubMed). There were no limits as to language or publication media. The latest search strategy was conducted in December 2011. It included randomized clinical trials that evaluated the use of platelet-rich plasma as coadjuvant medication to accelerate union of long bones (acute fractures, pseudoarthrosis and bone defects). The outcomes of interest for this review include bone regeneration, adverse events, costs, pain, and quality of life. The authors selected eligible studies, evaluated the methodological quality, and extracted the data. It was not possible to perform quantitative analysis of the grouped studies (meta-analyses).

**Results:**

Two randomized prospective clinical trials were included, with a total of 148 participants. One of them compared recombinant human morphogenic bone protein-7 versus platelet-rich plasma for the treatment of pseudoarthrosis; the other evaluated the effects of three coadjuvant treatments for union of valgising tibial osteotomies (platelet-rich plasma, platelet-rich plasma plus bone marrow stromal cells, and no coadjuvant treatment). Both had low statistical power and moderate to high risk of bias.

**Conclusion:**

There was no conclusive evidence that sustained the use of platelet-rich plasma as a coadjuvant to aid bone regeneration of fractures, pseudoarthrosis, or bone defects.

## RESUMO

**Objetivo:**

Avaliar a efetividade do uso de plasma rico em plaquetas como coadjuvante para a consolidação óssea de ossos longos.

**Métodos:**

A estratégia de busca abrangeu a *Cochrane Library* (via Central) e o MEDLINE (via PubMed). Não houve restrições de idioma ou meios de publicações. A última estratégia de busca foi realizada em dezembro de 2011. Foram incluídos ensaios clínicos randomizados que avaliaram o uso do plasma rico em plaquetas como medicação coadjuvante para acelerar a consolidação dos ossos longos (fraturas agudas, pseudoartroses e defeitos ósseos). Os desfechos de interesse para esta revisão compreenderam: consolidação óssea, eventos adversos, custos, dor e qualidade de vida. Os autores selecionaram os estudos elegíveis, avaliaram a qualidade metodológica e extraíram os dados. Não foi possível realizar análise quantitativa dos estudos agrupados (meta-análises).

**Resultados:**

Foram incluídos dois ensaios clínicos prospectivos randomizados, envolvendo um total de 148 participantes. Um deles comparou proteína morfogenética óssea recombinante humana 7 *versus* PRP para o tratamento de pseudoartroses; o outro avaliou os efeitos de três tratamentos coadjuvantes para a consolidação de osteotomias valgizantes da tíbia (plasma rico em plaquetas, plasma rico em plaquetas mais células estromais da medula óssea e sem tratamento coadjuvante). Ambos possuíam baixo poder estatístico e moderado a alto risco de viés.

**Conclusão:**

Não houve evidências conclusivas que sustentassem o uso de plasma rico em plaquetas como coadjuvante para auxiliar a consolidação óssea de fraturas, pseudoartrose ou defeitos ósseos.

## INTRODUCTION

Platelet-rich plasma (PRP) is a product derived from autologous blood, and its preparation is intended to obtain a high platelet concentration in a small volume of plasma. Both plasma and its preparation contain growth factors that play a role during the initial phase of healing and bone regeneration^(1,2)^. The primary growth factors involved in bone regeneration are platelets (platelet-derived growth factor – PDGF), transforming growth factor beta – TGF-β, insulin-like growth factor-1 – IGF-1, and the epidermal growth factor – EGF^(3,4)^. Nevertheless, the mechanism of action of these factors is not totally clear^(3,5)^.

PRP is obtained by centrifugation of autologous blood of the patients. The result of this centrifugation is a large concentration of platelets in a small volume of plasma. There are many methods for obtaining PRP, each one with specific properties as to capacity of concentration of the platelets and release process of certain growth factors. In order for PRP to have greater efficacy, the ideal concentration of platelets should be roughly 1,000,000*μ*L in a standard aliquot of 6mL^(6)^.

Therapies that use PRP may be utilized as coadjuvants in various interventions of oral and maxillofacial and orthopedic specialties, with the potential of accelerating bone regeneration and preventing pseudoarthrosis^(1)^. Preparations of PRP have been used since the beginning of the 1990s, and their clinical benefits were initially reported in oral and maxillofacial surgeries; however, increased commercial incentives of pharmaceutical industries, especially in sports medicine, have led to the popularization of these therapies in a disorganized and non-standardized manner^(7)^.

Autologous bone grafts combined with PRP have shown positive results in accelerating bone regeneration in animal models^(8–10)^. However, other animal studies concluded that the use of PRP in combination with heterologous bone grafts do not bring results superior to the use of autologous grafting alone^(11,12)^. The contradictory or inconclusive results on the effectiveness of PRP may be related to the wide variation in obtaining PRP.

As to the studies that evaluated the use of PRP for oral and maxillary surgery, a systematic review of Oral Health from the Cochrane Collaboration^(13)^, with level of evidence 1A as per the classification proposed by the Center for Evidence-Based Medicine, in Oxford (United Kingdom), found four studies, totalizing 114 patients (latest strategic search in January 2010). The studies assessed the efficacy of PRP use as coadjuvant of bone grafts for the elevation of the maxillary sinus. Results of this review demonstrated that there are no statistically significant differences between the groups of patients who received PRP as coadjuvant and those who did not receive it relative to clinical outcomes, failure in procedures, and complications^(13)^.

A narrative review reported that there is inconclusive evidence and limited studies that evaluated the use of PRP for long bone regeneration^(14)^. This review intends to evaluate the best evidence in literature of the studies that covered the use of PRP as coadjuvant for bone regeneration.

## OBJECTIVE

To evaluate the effectiveness of studies that address the use of PRP as a coadjuvant for long bone regeneration (acute fractures, pseudoarthrosis, and bone defects).

## METHODS

### Types of studies included

Systematic review studies, randomized or quasi-randomized controlled trials (method of patient allocation not totally randomized; examples: date of birth, hospital registration number, alternation), which evaluated the use of PRP as a coadjuvant for bone regeneration were included.

There was no restriction as to the language of the studies included; articles submitted in another language, different from Portuguese or English, were translated.

### Types of participants

Studies that evaluated adult patients with diagnoses of fractures, pseudoarthrosis, or bone defects of long bones were considered for inclusion.

### Types of intervention

The interventions evaluated were all studies that assessed the use of PRP as a coadjuvant. The primary comparisons of interest consisted of PRP *versus* placebo, autologous or heterologous bone graft, and no coadjuvant treatment.

### Types of outcomes evaluated

The outcomes of interest for this review were bone regeneration, adverse events, costs, pain, and quality of life.

### Electronic searches

The databases used were MEDLINE via PubMed (1966 until December, 2011) and the Cochrane Central Registry of Clinical Trials (CENTRAL; The Cochrane Library 2011, volume 12). The surveys also covered the protocols of current ongoing trials and those recently completed in Current Controlled Trials (http://www.controlled-trials.com/isrctn) and in the WHO International Registry of Clinical Trials (http://apps.who.int/trialsearch). There were no restrictions based on language or status of the publication.

Keywords and their synonyms were used in the search. On MEDLINE, the two initial phases of an ideal search^(15)^ were combined with the specific search subject, as per search strategies presented in the Cochrane Library.

### Search strategies

The objective of the strategy was to find randomized and quasi-randomized clinical trials, and systematic reviews of randomized clinical trials.

### MEDLINE (PubMed)

((Platelet-Rich Plasma [mh] OR Blood Platelets [mh] OR (platelet rich [tw] AND (plasma [tw] OR therap$ [tw] OR fibrin [tw])) OR PRP [tw] OR platelet plasma [tw] OR platelet gel [tw] OR platelet concentrate [tw]) AND (Fracture Healing [mh] OR Fracture Fixation [mh] OR Bone Regeneration [mh] OR Fractures, Bone [mh] OR Bone Remodeling [mh] OR Fractures, malunited [mh] OR Fractures, ununited [mh] OR fractur$ [tw])) AND (meta-analysis [pt] OR randomized controlled trial [pt] OR controlled clinical trial [pt] OR randomized controlled trials [mh] OR random allocation [mh] OR double-blind method [mh] OR single-blind method [mh] OR clinical trial [pt] OR clinical trials [mh] OR (“clinical trial” [tw]) OR ((singl* [tw] OR doubl* [tw] OR trebl* [tw] OR tripl* [tw]) AND (mask* [tw] OR blind* [tw])) OR (placebos [mh] OR placebo* [tw] OR random* [tw] OR research design [mh:noexp]) NOT (animals [mh] NOT human [mh]))

### The Cochrane Library (Wiley Interscience)

#1 MeSH descriptor Blood Platelets, this term only
#2 MeSH descriptor Platelet-Rich Plasma, this term only
#3 “platelet rich”: ti,ab,kw
#4 (PRP or “platelet plasma” or “platelet gel” or “platelet concentrate”): ti,ab,kw
#5 (#1 OR #2 OR #3 OR #4)
#6 MeSH descriptor Bone Remodeling, this term only
#7 MeSH descriptor Bone Regeneration, this term only
#8 MeSH descriptor Fractures, Bone, this term only
#9 MeSH descriptor Fracture Healing, this term only
#10 MeSH descriptor Fractures, Ununited explode all trees
#11 MeSH descriptor Fractures, Malunited, this term only
#12 MeSH descriptor Fracture Fixation, this term only
#13 fractur*: ti,ab,kw
#14 (#6 OR #7 OR #8 OR 9 OR #10 OR #11 OR
#12 OR #13)
#15 (“long bone” or “long-bone”):ti,ab,kw
#16 (#14 AND #15)
#17 (#5 AND #16)

### Evaluation of the methodological quality of the studies

The methodological quality of the studies included was evaluated subjectively, according to the evaluation tool proposed by the Cochrane Collaboration^(16)^. The domains measured were: (a) sequence of randomization generation; (b) allocation concealment; (c) blinding of patients and researchers; (d) blinding of outcome evaluators; (e) incomplete data; (f) selective outcome report; (g) other bias sources.

Domain analysis was judged in a subjective manner as low risk, uncertain risk, or high risk of bias. All disagreements were discussed and agreed upon among the authors.

## RESULTS

The search strategy in both databases found 105 references, but only 2 studies were relevant for the clinical question^(17,18)^ (Chart 1). No systematic review was found with level of evidence 1A, as per the classification proposed by the Center for Evidence-Based Medicine (Oxford, United Kingdom).

**Chart 1:** Characteristics of the studies included

Study	Method/Population	Interventions/Outcomes	Results/Conclusions	Limitations
Calori 2008^(17)^	Randomized clinical trial	Interventions: Group 1: rhBMP-7; Group 2: PRP.	Clinical and radiographic consolidation: 86.7% rhBMP-7 versus 68.3% PRP (P=0.016);	Bias risk: Randomization generation sequence: low bias risk; Allocation concealment: high bias risk; Blinded patients and researchers: high bias risk; Blinding of outcome evaluators: high bias risk; Incomplete data: uncertain bias risk; Selective report of outcomes: uncertain bias risk; Other bias sources: low bias risk.
	120 patients with atrophic pseudarthrosis of long bones (femur, tibia, humerus, ulna or radius).	Primary: clinical and radiographic consolidation. Secondary: complications and pain.	Pain: no statistically significant differences;	
	Group rhBMP-7: 60 patients (28 women) - mean age: 44 years; Group PRP: 60 patients (25 women) – mean age: 41 years.	Follow-up: 1, 3, 6, 9 and 12 months.	Complications: no statistically significant differences.	
			Conclusion: results favorable for the use of rhBMP-7 when compared with PRP for the outcome of bone consolidation.	
				Study with moderate/high methodological bias risk subjective criteria and with low statistical power.
Dallari 2007^(18)^	Randomized clinical trial	Interventions: Group A: lyophilized bone graft plus PRP; Group B: lyophilized bone graft plus PRP and bone marrow stromal cells; Group C: only lyophilized bone graft.	There were no statistically relevant differences between the intervention for functional clinical outcomes; Patients from Group B presented with an osteogenic potential and graft integration significantly higher than the other groups. However, there were also significant differences when comparing Group A with Group C.	Bias risk: Randomization generation sequence: low bias risk; Allocation concealment: high bias risk; Blinding of patients and researchers: high bias risk; Blinding of outcome evaluators: low bias risk; Incomplete date: uncertain bias risk; Selective report of the outcomes: uncertain bias risk; Other bias sources: low bias risk.
	28 patients submitted to valgising tibial osteotomy. Group A: 9 patients submitted to bone graft plus PRP (5 women) – mean age: 46.6 years; Group B: 10 patients submitted to bone graft plus PRP and stromal cells (4 women) - mean age: 52.6 years; Group C: 9 patients submitted only to bone graft (5 women) – mean age: 54.7 years.	Primary: a) clinical – functional score of the Knee Society and, b) radiographic – integration of the graft; Secondary: histopathological evaluation – osteogenesis, angiogenesis and inflammation.	Complications: none of the three groups presented significant complications.	
		Follow-up: 6 and 12 weeks, 6 and 12 months.	Conclusions: PRP or PRP with stromal cells showed better osteointegration of the graft; nevertheless, these coadjuvants do not alter the functional results.	Study with moderate/high methodological bias risk subjective criteria and with low statistical power.

As a result of the search in records of ongoing clinical trial protocols and systematic reviews, three randomized clinical trial projects^(19–21)^ and one systematic review protocol were identified^(22)^ – all without preliminary results until the closing of this paper (Chart 2).

**Chart 2:** Ongoing studies

Author	Reference
Griffin et al.^(19)^	Griffin XL, Parsons N, Achten J, Costa ML. Warwick Hip Trauma Study: a randomised clinical trial comparing interventions to improve outcomes in internally fixed intracapsular fractures of the proximal femur. Protocol for the WHiT Study. BMC Musculoskelet Disord. 2010;11:184
Petrera et al.^(20)^	Petrera P, Moody W, Christensen M. Platelet Rich Plasma (PRP) in Total Knee Replacement: A Prospective, Randomized, Single-blind, Single-center Clinical Study to Evaluate the Effect of Platelet Rich Plasma (PRP) on Short-term Patient Outcomes Following Total Knee Replacement. WHO International Clinical Trial Registry. [cited 2011 Dec. 27]. Available from: http://clinicaltrials.gov/show/NCT00826098
Smith et al.^(21)^	Smith KK. Standard total knee arthroplasty using platelet rich plasma (PRP). WHO International Clinical Trial Registry. [cited 2011 Dec 27]. Available from: http://clinicaltrials.gov/show/NCT01075230
Griffin et al.^(22)^	Griffin XL, Wallace D, Parsons N, Costa ML. Platelet rich therapies for long bone healing in adults. Cochrane Database of Systematic Reviews 2011, Issue 12. Art. No.: CD009496

The clinical trial by Calori et al.^(17)^ was one of those included in this project. It is a randomized prospective trial that compared recombinant bone morphogenetic protein 7–rhBMP-7 with PRP for the treatment of pseudoarthrosis. The other trial, also randomized and prospective, was carried out by Dallari et al.^(18)^, and evaluated the effects of three coadjuvant treatments for the consolidation of valgising tibial osteotomies (correction of *genu varum*). The three groups of coadjuvant treatments were Group A, with lyophilized bone bank graft plus PRP gel; Group B, with lyophilized bone bank graft plus PRP gel and bone marrow stromal cells, and Group C, using only a lyophilized bone bank graft.

## DISCUSSION

This review included only two randomized clinical trials, involving a total of 148 participants. The studies included had low statistical power and moderate to high risk of methodological bias. It was not possible to carry out a quantitative analysis (meta-analysis) of the combination of the studies included. The data available were not grouped due to the considerable variation of the methods of treatment and to the heterogeneity of the studies and outcomes evaluated.

The search strategy was designed with the objective of locating all the possible studies with an adequate level of evidence (systematic reviews and randomized clinical trials). Effort was made to identify the primary relevant studies that evaluated the use of PRP in aiding bone regeneration; however, it is possible that a potential study might not have been included.

The two studies included did not allow a comprehensive review of the relative effectiveness of PRP use as a coadjuvant for bone regeneration. From these comparisons of treatment described in this review, it was not possible to obtain a high degree of evidence, due to the high risk of bias, the low statistical power, and the small number of studies included.

Calori et al.^(17)^ compared rhBMP-7 with PRP for the treatment of pseudoarthrosis. The results found showed superiority in the process of union (clinical and radiographic) in patients treated with rhBMP-7. Nevertheless, these results should be interpreted with caution, since the placebo intervention was not compared to the two interventions evaluated by the study; the follow-up of the patients lasted only 12 months; and the authors did not prospectively control the differences of the results relative to the impregnation of rhBMP-7 and PRP in bone grafts.

Dallari et al.^(18)^ compared three coadjuvant interventions (PRP, PRP plus bone marrow stromal cells, and no coadjuvant treatment) to lyophilized bone graft in patients submitted to valgising tibial osteotomy. The study showed no differences in the outcomes evaluated after one year; nevertheless, the short-term conclusions, related to the radiographic and histomorphometric data, encourage the use of PRP as a coadjuvant. This study showed important restrictions that should be considered when interpreting its results. Among the main restrictions, it is noted that the primary outcomes are not clearly reported, these are multiple analyses with no adjustment for the statistically significant outcomes, and the study shows low statistical power with type II error possibility in its results.

Other limitations inherent to both studies are relative to the absence of a description of the strategies that would avoid the primary risks of bias: risk of selection, in not describing allocation concealment; and risk of performance, in not describing the blinding of the participants and researchers.

The preparation of therapeutic doses of PRP is performed by means of the collection of the patient's autologous blood, plasma separation (blood centrifugation), and the application of PRP (with the growth factors) at the site of the lesion. The conflicting results among the orthopedic studies probably are due to non-standardization of the blood centrifugation processes. Literature has shown that there is no uniformity among the various methods of centrifugation and obtaining growth factors. Narrative reviews suggest that the different methods for obtaining PRP may result in different clinical effects for the patients^(23–25)^. From the results of these studies, the need for future comparative studies is evident, which would evaluate the innumerable methods of obtaining PRP, the different uses of coadjuvant coagulating factors, and the technique for selectively obtaining specific growth factors.

The contraindications for PRP are preexisting coagulopathies, active infection, and pregnancy, hypersensitivity to bovine thrombin, malignant neoplasms and metastatic tumors^(1)^. The studies included in this review did not report any relevant complications; only Calori et al.^(17)^ reported infection in five patients after the use of PRP.

Systemic complications related to the use of PRP are described in the medical literature; among the most common are infections inherent to any invasive interventions^(26–28)^. Other contradictory adverse events are possible induction of neoplastic diseases and muscle tissue fibrosis^(26)^; however, some studies concluded that there are not sufficient data to affirm that these complications are directly related to the use of PRP^(29,30)^. The performance of new studies that would evaluate the adverse events of PRP is necessary.

Recently the Food and Drug Administration (FDA) approved various methods and centrifuges for obtaining the PRP preparations. The centrifuge devices for producing PRP should be used in a laboratory or at the site of patient care^(31)^. Nevertheless, the use of PRP in clinical practice is not totally approved in some countries. In 2008, The National Institute for Health and Clinical Excellence (NICE) declared that there is insufficient evidence to allow the use of PRP in clinical routine, and that its use should be restricted only to clinical research^(32)^.

## CONCLUSION

There is no conclusive evidence that sustains the use of PRP as a coadjuvant to aid in the bone consolidation of fractures, pseudoarthrosis, or bone defects. There is immediate need for the preparation of randomized prospective clinical trials, with adequate methodological quality and high statistical power to investigate the effectiveness of PRP use as a coadjuvant in bone regeneration.
